# Clinical and Epidemiologic Characteristics of Mpox Cases, Dominican Republic, July 2022–February 2023

**DOI:** 10.3201/eid3105.241299

**Published:** 2025-05

**Authors:** Robert Paulino-Ramirez, Wilfredo Rafael Matias, Hector Lora-Rodríguez, Grey Benoît, Joel Ureña, Monica Thormann, Ronald Skewes-Ramm

**Affiliations:** Universidad Iberoamericana, Santo Domingo, Dominican Republic (R. Paulino-Ramirez, H. Lora-Rodriguez); Brigham and Women's Hospital, Boston, Massachusetts, USA (W.R. Matias); Massachusetts General Hospital, Boston (W.R. Matias); Ministry of Health, Santo Domingo (G. Benoît, M. Thormann, R. Skewes-Ramm); Ministry of Defense, Santo Domingo (J. Ureña)

**Keywords:** mpox, monkeypox virus, epidemiology, sexually transmitted infections, viruses, zoonoses, Dominican Republic

## Abstract

During July 2022–February 2023, mpox was confirmed in 71 of 283 suspected cases in the Dominican Republic; 32.4% of patients were women, and 22.5% children <10 years of age. We found differences in transmission compared with global trends, emphasizing the need for continued surveillance, diagnostics, and public health interventions.

Mpox is a zoonotic disease caused by the monkeypox virus (MPXV), which belongs to clade I (formerly Congo Basin clade) or clade II (formerly West Africa clade) (*1*). First identified in humans in the Democratic Republic of the Congo, mpox has become a global public health concern (*2*). As of December 2024, a total of 124,753 cases and 272 deaths have been reported in 128 countries; 54% of cases occurred in the Americas (*3*). Mpox spreads through close physical contact and fomites (*4*). Although sexual contact played a key role in the 2022–2023 mpox outbreak, nonsexual transmission also occurs, especially in children (*5*). In Latin America and the Caribbean, MPXV has circulated with varying disease outbreak intensity (*6*). 

In the Dominican Republic, the first confirmed mpox case was reported in July 2022. We reviewed clinical and epidemiologic characteristics of reported cases to elucidate the epidemic in the Dominican Republic and guide public health efforts. 

We conducted a retrospective review of the Dominican Republic’s national mpox database, which contains epidemiologic data collected by the Ministry of Public Health. Mpox is a reportable disease in the Dominican Republic, requiring healthcare workers to complete forms for suspected and confirmed cases, which are then uploaded to the national database. We analyzed data representing the initial outbreak period during July 2022–February 2023 and defined cases as suspected, probable, or confirmed by using World Health Organization criteria (*7*). Medical authorities interviewed patients suspected of having mpox and collected sociodemographic and clinical information; samples for MPXV testing were taken from lesions, exudates, crusts, and blood (if no rash was present). The National Public Health Laboratory conducted sample testing by MPXV PCR, targeting G2R and C3L loci according to World Health Organization guidelines (*8*); a positive PCR result confirmed diagnosis of mpox. After confirmation, Ministry of Public Health epidemiologists conducted mandatory reporting, contact tracing, and household contact testing. We categorized suspected cases as confirmed or negative. We used R version 4.3.2 (The R Project for Statistical Computing, https://www.r-project.org) for analyses. 

After the first mpox case was identified (July 2022), cases peaked in September and declined thereafter (Figure). Among 283 suspected cases, 71 (25.1%) were confirmed. Among confirmed mpox patients, 48 (67.6%) were male and 23 (32.4%) female; most (49.3%) male patients were 20–39 years of age. Sixteen (22.5%) confirmed mpox patients were children <10 years of age. Most (95.8%) confirmed mpox patients were born in the Dominican Republic; most (n = 39) cases occurred in the O Metropolitana region, encompassing Santo Domingo (Appendix Figure). We compiled sociodemographic and clinical characteristics (Table; Appendix Table). Most (90.1%) confirmed mpox patients manifested a rash, and 73.2% reported fever (>37.9°C; self-reported with or without a thermometer or measured by the person conducting household visits).

**Figure:**
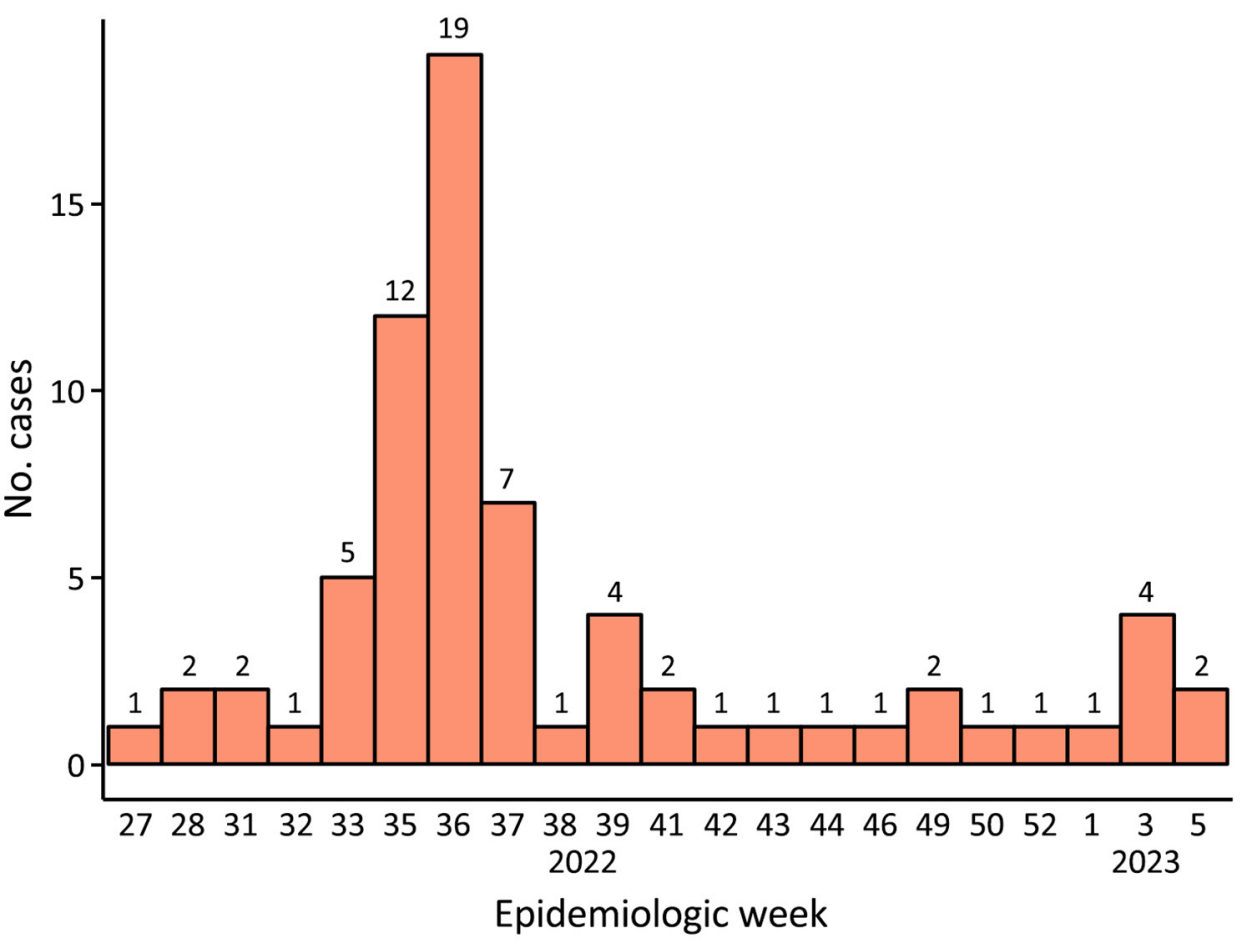
Number of confirmed mpox cases, by epidemiologic week, in study of clinical and epidemiologic characteristics of mpox, Dominican Republic, July 2022–February 2023. Numbers at the top of each bar indicate the actual number of cases for that week.

**Table:** Clinical and epidemiologic characteristics of suspected mpox cases in the Dominican Republic, July 2022–February 2023*

Patient characteristics	Confirmed cases	Negative cases
Total	71 (100.0)	212 (100.0)
Sex		
M	48 (67.6)	124 (58.5)
F	23 (32.4)	88 (41.5)
Age range, y		
0–9	16 (22.5)	55 (25.9)
10–19	6 (8.5)	22 (10.4)
20–29	19 (26.8)	41 (19.3)
30–39	16 (22.5)	45 (21.2)
40–49	4 (5.6)	15 (7.1)
50–59	7 (9.9)	15 (7.1)
>60	3 (4.2)	19 (9.0)
Country of birth		
Dominican Republic	68 (95.8)	202 (95.3)
Other†	3 (4.2)	10 (4.7)
Highest education level		
None	10 (14.1)	38 (17.9)
Elementary school	8 (11.3)	23 (10.9)
Middle school	7 (9.9)	17 (8.0)
High school	14 (19.7)	47 (22.2)
College or technical school	11 (15.5)	24 (11.3)
Missing	21 (29.6)	63 (29.7)
Health region of residence		
I Valdesia	13 (18.3)	19 (9.0)
II Cibao Norte	11 (15.5)	25 (11.8)
III Cibao Nordeste	0	11 (5.2)
IV Enriquillo	0	10 (4.7)
O Metropolitana	39 (54.9)	122 (57.5)
V Este	6 (8.5)	11 (5.2)
VI Del Valle	0	2 (0.9)
VII Cibao Occidental	1 (1.4)	9 (4.2)
VIII Cibao Central	1 (1.4)	3 (1.4)
Health insurance type		
Private	23 (32.4)	55 (25.9)
Government subsidized	8 (11.3)	20 (9.4)
No reported insurance	11 (15.5)	53 (25.0)
Unknown	29 (40.8)	84 (39.6)
Time from symptom onset to seeking care, d (range)	5.0 (2.5–7.0)	4.0 (2.0–7.0)
Underlying illness		
Chronic diseases‡	5 (7.0)	8 (3.8)
HIV/AIDS	7 (9.9)	3 (1.4)
Malnutrition	0 (0.0)	2 (0.9)
Obesity/overweight	0 (0.0)	3 (1.4)
None	29 (40.8)	99 (46.7)
Missing	30 (42.3)	97 (45.8)

*Values are no. (%) except as indicated. †Includes Germany, Belgium, United States, Haiti, Italy, Democratic Republic of Congo, Switzerland, and Venezuela. ‡Includes diabetes, chronic respiratory disease, hypertension, or other cardiovascular disease.

The Dominican Republic’s mpox outbreak revealed unique epidemiologic patterns compared with other outbreaks; a higher percentage of women (32.4%) and children <10 years of age (22.5%) was observed. The patterns resemble those in endemic countries in Africa but contrast with global trends during the 2022–2023 outbreak, where most cases occurred in adult men who have sex with men (MSM) and only 3.6% of women and 1.1% of children <17 years of age globally (*9*). This divergence reflects either genuine differences in transmission dynamics or underreporting among MSM because of stigma and homophobia, which are common in the Dominican Republic and heightened by the intersectionality of HIV (*10*). Societal stigma against MSM in the Dominican Republic might influence sexual networks, leading to concurrent relationships with both men and women, promoting mpox transmission into heterosexual households. Within households, mpox might spread via close contact, caregiving, and shared personal items, contributing to more cases among women and children. Those findings highlight the need for comprehensive public health approaches, including stigma reduction and fostering community trust. Further research is needed to clarify transmission and guide interventions.

Positive mpox cases were clinically similar to negative cases; most patients experienced a rash and fever. Although using rash and fever as screening criteria assists case identification, their lack of specificity poses challenges, particularly in settings with limited diagnostic capabilities. Combining clinical criteria with exposure history or risk profiles might improve testing efficiency in diagnostic-constrained settings. Expanding access to diagnostics and developing easy-to-use tests for resource-limited settings are critical for accurate case identification and optimized resource allocation.

The first limitation of our study is that data collected during outbreak investigations likely underestimate true incidence. Second, because of the evolving nature of the outbreak, comprehensive data, including sexual orientation, were not available.

In conclusion, we describe the initial mpox outbreak during July 2022–February 2023 in the Dominican Republic; the percentage of infected women and children was higher than expected compared with the global outbreak predominantly affecting MSM. Although the country successfully implemented mandatory reporting and vaccine deployment, challenges remain, such as incomplete data capture, limited access to PCR, and barriers to healthcare access. Addressing those gaps will be essential to improve outbreak detection and prepare for future mpox outbreaks.

Appendix Additional information for clinical and epidemiologic characteristics of mpox cases, Dominican Republic, July 2022–February 2023.

## References

[1] Mitjà O, Ogoina D, Titanji BK, Galvan C, Muyembe JJ, Marks M, et al. Monkeypox. Lancet. 2023;401:60–74. 10.1016/S0140-6736(22)02075-X 36403582 PMC9671644

[2] Ladnyj ID, Ziegler P, Kima E. A human infection caused by monkeypox virus in Basankusu Territory, Democratic Republic of the Congo. Bull World Health Organ. 1972;46:593–7. 4340218 PMC2480792

[3] World Health Organization. Mpox: multi-country external situation report no. 46, published 28 January 2025 [cited 2025 Jan 30]. https://cdn.who.int/media/docs/default-source/documents/emergencies/mpox-multi-country-external-situation-report-no.-46.pdf

[4] Alakunle EF, Okeke MI. Monkeypox virus: a neglected zoonotic pathogen spreads globally. Nat Rev Microbiol. 2022;20:507–8. 10.1038/s41579-022-00776-z 35859005 PMC9297671

[5] Low N, Bachmann LH, Ogoina D, McDonald R, Ipekci AM, Quilter LAS, et al. Mpox virus and transmission through sexual contact: Defining the research agenda. PLoS Med. 2023;20:e1004163. 10.1371/journal.pmed.1004163 36649325 PMC9888714

[6] Quispe AM, Castagnetto JM. Monkeypox in Latin America and the Caribbean: assessment of the first 100 days of the 2022 outbreak. Pathog Glob Health. 2023;117:717–26. 10.1080/20477724.2023.2201979 37057838 PMC10614714

[7] World Health Organization. Surveillance, case investigation and contact tracing for mpox ‎‎(monkeypox)‎. Interim guidance, ‎20 March 2024‎ [cited 2024 Nov 27]. https://iris.who.int/bitstream/handle/10665/376306/WHO-MPX-Surveillance-2024.1-eng.pdf

[8] Pan American Health Organization, World Health Organization. Laboratory guidelines for the detection and diagnosis of monkeypox virus infection [cited 2024 Aug 24]. https://www.paho.org/en/documents/laboratory-guidelines-detection-and-diagnosis-monkeypox-virus-infection

[9] Laurenson-Schafer H, Sklenovská N, Hoxha A, Kerr SM, Ndumbi P, Fitzner J, et al.; WHO mpox Surveillance and Analytics team. Description of the first global outbreak of mpox: an analysis of global surveillance data. Lancet Glob Health. 2023;11:e1012–23. 10.1016/S2214-109X(23)00198-5 37349031 PMC10281644

[10] Yigit I, Paulino-Ramírez R, Waters J, Long DM, Turan JM, Budhwani H. A moderated mediation analysis of HIV and intersectional stigmas and antiretroviral adherence in people living with HIV in the Dominican Republic. AIDS Behav. 2024;28:3258–69. 10.1007/s10461-024-04425-9 38916689 PMC11524671

