# Supplementary material for: Clinical and Epidemiologic Characteristics of Mpox Cases, Dominican Republic, July 2022–February 2023
Source: Emerg Infect Dis. 2025 May;31(5):1060–2. doi: 10.3201/eid3105.241299 (PMC12044227; doi:10.3201/eid3105.241299)
Supplement: Appendix — Additional information for clinical and epidemiologic characteristics of mpox cases, Dominican Republic, July 2022–February 2023. [file 24-1299-Techapp-s1.pdf]

Article DOI: https://doi.org/10.3201/eid3105.241299

EID cannot ensure accessibility for supplementary materials supplied by authors. Readers who have difficulty accessing supplementary content should contact the authors for assistance.

Clinical and Epidemiologic Characteristics of Mpox Cases, Dominican Republic, July 2022–February 2023

Appendix

Appendix Table. Signs, symptoms, and rash location in cases of suspected mpox in the Dominican Republic, July 2022–February 2023

Patient characteristics	Confirmed cases, no. (%)	Negative cases, no. (%)
Total no.	71 (100.0)	212 (100.0)
Skin rash	64 (90.1)	176 (83.0)
Skin rash location		
Head	8 (12.5)	15 (8.5)
Face	16 (25.0)	40 (22.7)
Mouth, lips, or oral mucosa	1 (1.6)	2 (1.1)
Neck	5 (7.8)	15 (8.5)
Upper extremities	14 (21.9)	49 (27.8)
Hands	7 (10.9)	17 (9.7)
Trunk	16 (25.0)	53 (30.1)
Lower extremities	12 (18.8)	41 (23.3)
Genitals	6 (9.4)	10 (5.7)
Perianal	5 (7.8)	8 (4.5)
Feet	4 (6.2)	15 (8.5)
Missing or unknown location	27 (42.2)	70 (39.8)
Pruritus	16 (22.5)	56 (26.4)
Fever	52 (73.2)	143 (67.5)
Malaise	26 (36.6)	59 (27.8)
Chills	10 (14.1)	14 (6.6)
Lymphadenopathy	6 (8.5)	9 (4.2)
Headache	23 (32.4)	59 (27.8)
Myalgia or arthralgia	12 (16.9)	50 (23.6)
Rectal pain	0 (0.0)	0 (0.0)
Rectal bleeding	1 (1.4)	1 (0.5)
Tenesmus	0 (0.0)	0 (0.0)
Proctitis	0 (0.0)	0 (0.0)
Pus or blood in stools	1 (1.4)	0 (0.0)
Nausea, vomiting, or loss of appetite	1 (1.4)	12 (5.7)
Abdominal pain	0 (0.0)	1 (0.5)
Conjunctivitis	1 (1.4)	2 (0.9)
Other signs and symptoms	3 (4.7)	0 (0.0)

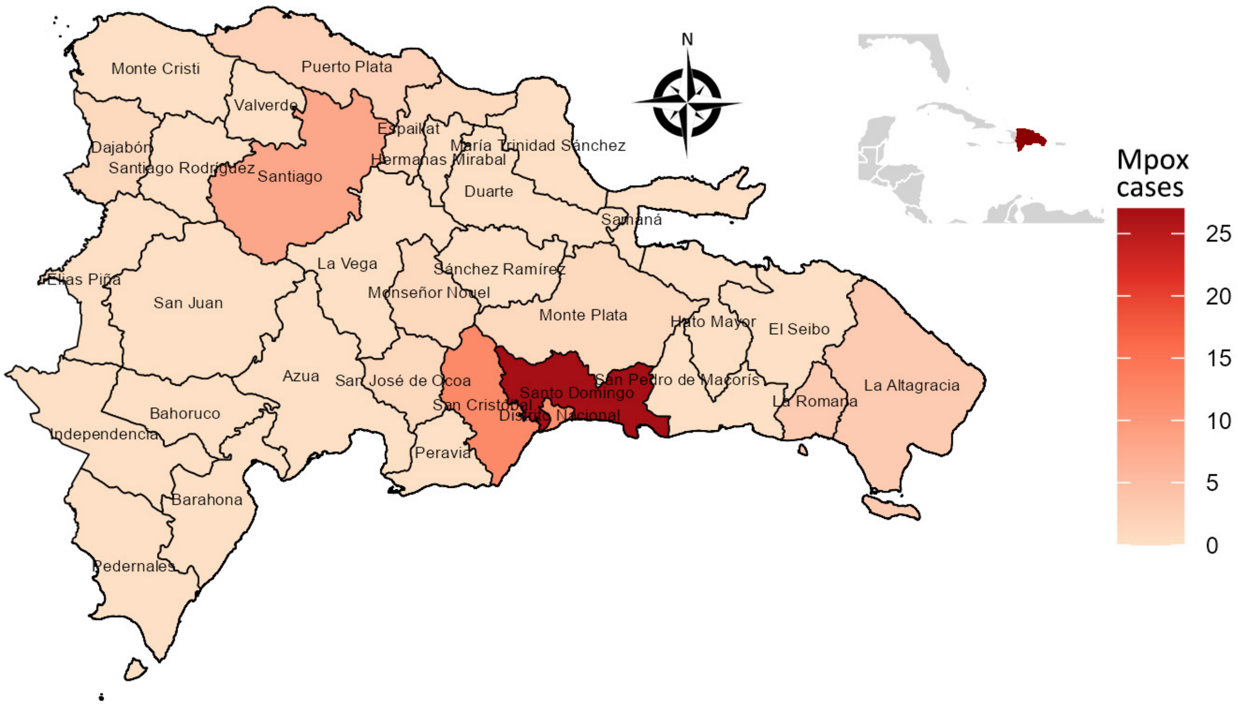

Appendix Figure. Geographic distribution of mpox cases in the Dominican Republic, July 2022–February 2023. Color gradient indicates the number of cases in each region of the country.
